# Structural Insights into Higher Order Assembly and Function of the Bacterial Microcompartment Protein PduA*

**DOI:** 10.1074/jbc.M114.569285

**Published:** 2014-05-29

**Authors:** Allan Pang, Stefanie Frank, Ian Brown, Martin J. Warren, Richard W. Pickersgill

**Affiliations:** From the ‡ School of Biological and Chemical Sciences, Queen Mary University of London, Mile End Road, London E1 4NS, United Kingdom and; the § School of Biosciences, University of Kent, Canterbury, Kent CT2 7NJ, United Kingdom

**Keywords:** Bacteria, Crystal Structure, Electron Microscopy (EM), Molecular Modeling, Synthetic Biology, Bacterial Microcompartment Protein PduA

## Abstract

Bacterial microcompartments are large proteinaceous assemblies that are found in the cytoplasm of some bacteria. These structures consist of proteins constituting a shell that houses a number of enzymes involved in specific metabolic processes. The 1,2-propanediol-utilizing microcompartment is assembled from seven different types of shell proteins, one of which is PduA. It is one of the more abundant components of the shell and intriguingly can form nanotubule-like structures when expressed on its own in the cytoplasm of *Escherichia coli*. We propose a model that accounts for the size and appearance of these PduA structures and underpin our model using a combinatorial approach. Making strategic mutations at Lys-26, Val-51, and Arg-79, we targeted residues predicted to be important for PduA assembly. We present the effect of the amino acid residue substitution on the phenotype of the PduA higher order assemblies (transmission electron microscopy) and the crystal structure of the K26D mutant with one glycerol molecule bound to the central pore. Our results support the view that the hexamer-hexamer interactions seen in PduA crystals persist in the cytoplasmic structures and reveal the profound influence of the two key amino acids, Lys-26 and Arg-79, on tiling, not only in the crystal lattice but also in the bacterial cytoplasm. Understanding and controlling PduA assemblies is valuable in order to inform manipulation for synthetic biology and biotechnological applications.

## Teaser

**Background:** PduA is a major shell protein in 1,2-propanediol utilization microcompartments.

**Results:** The profound influence of Lys-26 and Arg-79 on hexagonal tiling is revealed both in the crystal and *in vivo*.

**Conclusion:** Interactions seen in the crystal are important for sheet and nanotube assembly in the bacterial cytoplasm.

**Significance:** This work provides knowledge for the engineering of PduA for stability and porosity for synthetic biology applications.

## Introduction

Bacterial microcompartments (BMCs)^5^ are polyhedral cellular inclusions found in several bacterial species (1–3). BMCs were discovered in cyano- and chemotrophic bacteria by transmission electron microscopy (TEM) of thin sections and were at first mistaken for phage capsids, because they share similar size and shape (4). BMCs are, in fact, composed of a protein shell encapsulating the enzymes involved in carbon fixation (carboxysomes) (5) or of a metabolic pathway (metabolosomes) (6–10). The shell-forming proteins contain bacterial microcompartment domains (11). The majority of shell proteins consist of a single BMC domain (Pfam 00936) in each subunit and assemble into hexamers. Some shell proteins consist of a tandem repeat of the Pfam 00936 fold and form pseudohexameric trimers (12–14). It is plausible that the vertices are occupied by pentameric shell proteins (Pfam 03319).

The shell of the 1,2-propanediol utilization (Pdu) metabolosome is composed of seven shell proteins: PduA, PduB, PduJ, PduK, PduN, PduT, and PduU (1, 15, 16). Previous work on the synthesis of empty Pdu microcompartments showed that not all of these seven *pdu* genes encoding shell proteins are necessary for the formation of heterologous microcompartments in *Escherichia coli* (17). PduA, PduB, PduB′, PduJ, PduK, and PduN were identified as the minimum shell components to form a non-aberrant empty Pdu microcompartment. Among the shell proteins, PduA is a major shell component of the Pdu metabolosome shell (18). PduA has been shown to interact with the majority of the other shell proteins and, thus, could potentially act as a scaffold for the assembly of the microcompartment (17). Deletion of PduA from a construct harboring the minimum number of genes for the formation of empty BMCs resulted in the formation of elongated filamentous structures that no longer resembled bacterial microcompartments (17). Interestingly, when PduA is overproduced alone in *E. coli*, it is able to form regular nanotube-like structures within the cytoplasm of the cell. A serendipitously obtained PduA construct (PduA*) with an extra 23 residues at the C terminus of the protein resulted in a more soluble protein and greater density of the nanostructures, giving greater visual impact in micrographs (17). In longitudinal sections through *E. coli* cells producing PduA*, thin parallel filamentous structures were observed, whereas in cross-sections, honeycomb-like structures were observed (17).

**FIGURE 1.**
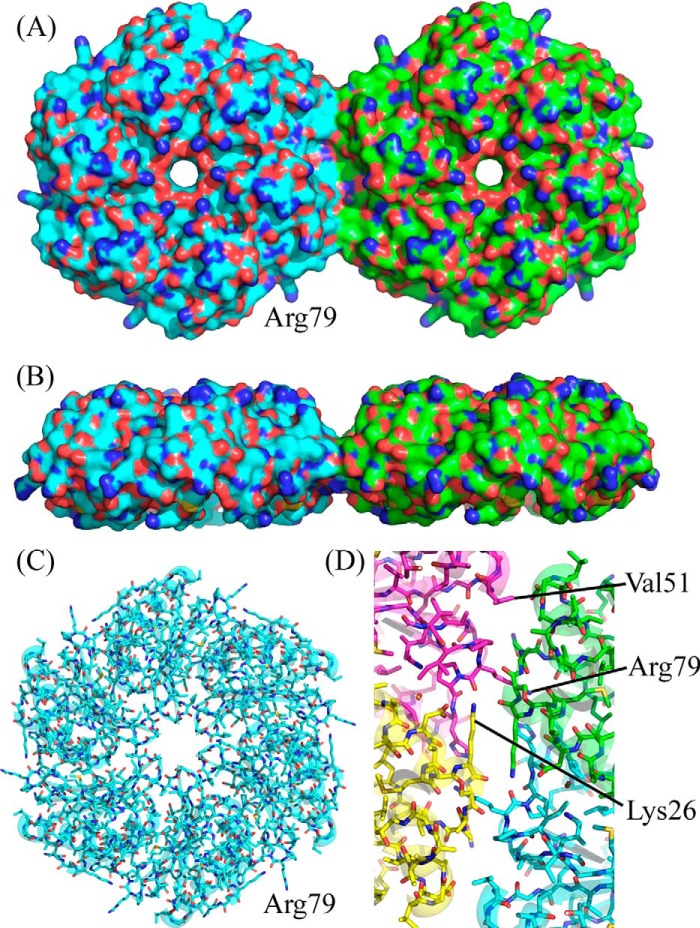
**Crystal structure of native PduA (Protein Data Bank accession code 3NGK) (19).** Six bacterial microcompartment protein PduA subunits form a hexamer with strict 6-fold rotational symmetry, and adjacent hexamers tile a surface. *A*, a surface representation of two PduA hexamers, one with *cyan* carbon atoms and one with *green*. One hexamer-hexamer interface is represented; this interaction is repeated to form a sheet of molecules. Arginine 79 can be seen protruding from each subunit of the hexamer for which adjacent hexamers are not drawn. *B*, the same two hexamers as in *a* but now rotated about the *x axis* by 90° to illustrate the narrow strip of the edge of the hexamer involved in the hexamer-hexamer interaction. *C*, *stick representation* of a single PduA hexamer; the three residues chosen for mutation to probe hexamer-hexamer interactions can be clearly seen: Lys-26, Val-51, and Arg-79. *D*, subunit *colored representation* of the hexamer-hexamer interaction surface showing how one edge of the hexamer is constructed of two adjacent PduA subunits. Approximately 1,200 Å^2^ are buried at the interface between two adjacent hexamers. Arg-76 and Lys-26 contribute most to the buried (accessible) surface area, contributing 140 and 77 Å^2^, respectively. Val-51 contributes a more modest but not insignificant 57 Å^2^.

The crystal structure of PduA from *Salmonella enterica* (Protein Data Bank accession code 3NGK) (19) revealed that the PduA subunits pack closely together to form a biologically authentic hexamer. Of the ∼20,400-Å^2^ total surface of the isolated PduA subunit, ∼9,600 Å^2^ are buried in the hexamer. The native PduA hexamers in the crystal have strict 6-fold rotational symmetry (Fig. 1) and tile within the crystal lattice (of space group P622) with adjacent hexamers separated by 67.2 Å (the *a* axis of the cell). Four PduA subunits come together to form the edge between two hexamers, and together they bury only ∼1,200 Å^2^ (Fig. 1*D*). It was suggested that although this is a narrow edge (Fig. 1*B*), the tiling of hexamers is probably closely similar to that occurring on a facet of the microcompartment (17).

Here, we suggest a model for the filamentous nanostructure produced by PduA* in the *E. coli* cytoplasm and provide mutational evidence in support of the model. We use a combination of mutagenesis, TEM, and crystallography to study morphological changes of the nanostructures resulting from changes of hexamer-hexamer interactions. We provide evidence that the interactions between PduA hexamers seen in the crystal persist in the bacterial cytoplasm. Moreover, we present the crystal structure of a PduA variant that has a substrate analog bound to the central pore.

## EXPERIMENTAL PROCEDURES

### 

#### 

##### Bioinformatics and Modeling

The interface between PduA hexamers in the crystal was analyzed using CCP4 programs PDBSET and AREAIMOL (20). PyMOL (21) was used for visualization and to produce the figures of molecules. Sequence conservation across several species of PduA was assessed using ClustalW (22). SymmDock (23, 24) was used to generate symmetry-related hexamers, which were then manually docked to form nanotubes using PyMOL (21).

##### Generation of PduA* Mutants

The sequence of *Citrobacter freundii* PduA is essentially the same as that from *S. enterica*. The only difference is that the *C. freundii* protein is one residue shorter; it lacks the C-terminal glutamine of *S. enterica*. The cloning of PduA* into pET3a has been described previously (17). PduA* is a more soluble form of *C. freundii* PduA with Arg-93 instead of Ser-93 and 23 additional C-terminal residues: LVKDPAANKARKEAELAAATAEQ. The Stratagene QuikChange protocol was used to generate five mutants, K26A, K26D, V51A, V51D, and R79A, using mutagenic primers. The correct sequence of the constructs was verified by DNA sequencing (Eurofins). PduA* K26D was subcloned into pET14b to facilitate PduA* K26D overproduction as a fusion protein with an N-terminal His_6_ tag in *E. coli*. In this paper, PduA will be used in place of the PduA* designation.

##### Transmission Electron Microscopy

*E. coli* BL21(DE3) pLysS cells were transformed with PduA and the five PduA mutant constructs. Bacteria cells were grown in 50 ml of lysogeny broth containing 100 mg/liter ampicillin with aeration at 37 °C. Upon reaching an *A*_600_ of 0.8, protein production was induced with 0.4 mm isopropyl β-d-thiogalactoside, and the cultures were incubated by shaking overnight at 18 °C. Harvested cells were resuspended in 2 ml of fixative consisting of 2.5% glutaraldehyde in phosphate-buffered saline (PBS). The cells were pelleted and washed twice with PBS to remove traces of the fixing solution. Cells were then stained for 1 h in 1% osmium tetroxide and washed with PBS before dehydration. Dehydration was carried out by placing the samples into a solvent gradient: 60% industrial methylated spirit overnight, 90% industrial methylated spirit for 15 min, 100% industrial methylated spirit for 15 min, and 100% dried ethanol twice for 2 h. The cells were embedded by first incubating them overnight in 30% agar low viscosity resin in dried ethanol and then embedding them for 180 min in 100% agar low viscosity resin that was constituted for a block with medium hardness (three changes of resin). The samples were placed in 0.5-ml embedding tubes, centrifuged for 5 min at 4,000 × *g* to concentrate the cells to the tip of the tube, and incubated at 60 °C overnight to polymerize. Specimens were thin sectioned with a diamond knife on an RMC MT-6000-XL ultramicrotome, collected on copper grids, and post-stained with 5% uranyl acetate for 30 min at 60 °C and 0.1% lead citrate for 10 min at room temperature. Sections were then observed and photographed with a JEOL-1230 transmission electron microscope.

##### Production, Purification, and Crystallization of PduA K26D

BL21*(DE3) pLysS harboring pET14b-PduA K26D were cultured for 21 h at 28 °C in 1 liter of 2× YT medium supplemented with 100 mg/liter ampicillin and 35 mg/liter chloramphenicol. Cells were harvested by centrifugation (10 min, 4,000 × *g*) and resuspended in a total volume of 30 ml of binding buffer (50 mm Tris-HCl, pH 8.0, 0.5 m NaCl, 10 mm imidazole). Cells were lysed by sonication, and cell debris was removed by centrifugation (35,000 × *g* for 20 min). The recombinant protein was purified using IMAC. The bound fraction was washed with increasing amounts of imidazole and eluted in 400 mm imidazole. The histidine tag was cleaved off of the purified protein by overnight incubation with thrombin. PduA K26D was further purified, and thrombin was removed by passing it over a size exclusion column (Superdex 200 Global 10/30) equilibrated with 50 mm Tris-HCl, pH 8.0, 100 mm NaCl. PduA K26D crystals were grown by hanging drop vapor diffusion. Type I crystals were harvested from protein drops (2.9 mg/ml) equilibrated against a reservoir of 1.3 m sodium citrate tribasic dihydrate, 0.1 m sodium HEPES, pH 7.9. The reservoir for type II crystals was 1.0 m sodium citrate tribasic dihydrate and 0.1 m Tris at pH 8.5, and the protein used was at 6.3 mg/ml. Reservoir augmented with 15% glycerol was used as a cryoprotectant, and x-ray diffraction data to 1.72 and 1.93 Å resolution were collected from the two different crystal forms at the Diamond Light Source (I03) using the PILATUS 6 m-F pixel detector. Data were processed using XDS (25) and XDSME and scaled using SCALA (26). The reduced data were analyzed using MOLREP (27), REFMAC (28), and COOT (29), and the quality of the final model was assessed using PROCHECK (30).

## RESULTS

### 

#### 

##### Modeling of Higher Order PduA Structures Observed in TEM

**FIGURE 2.**
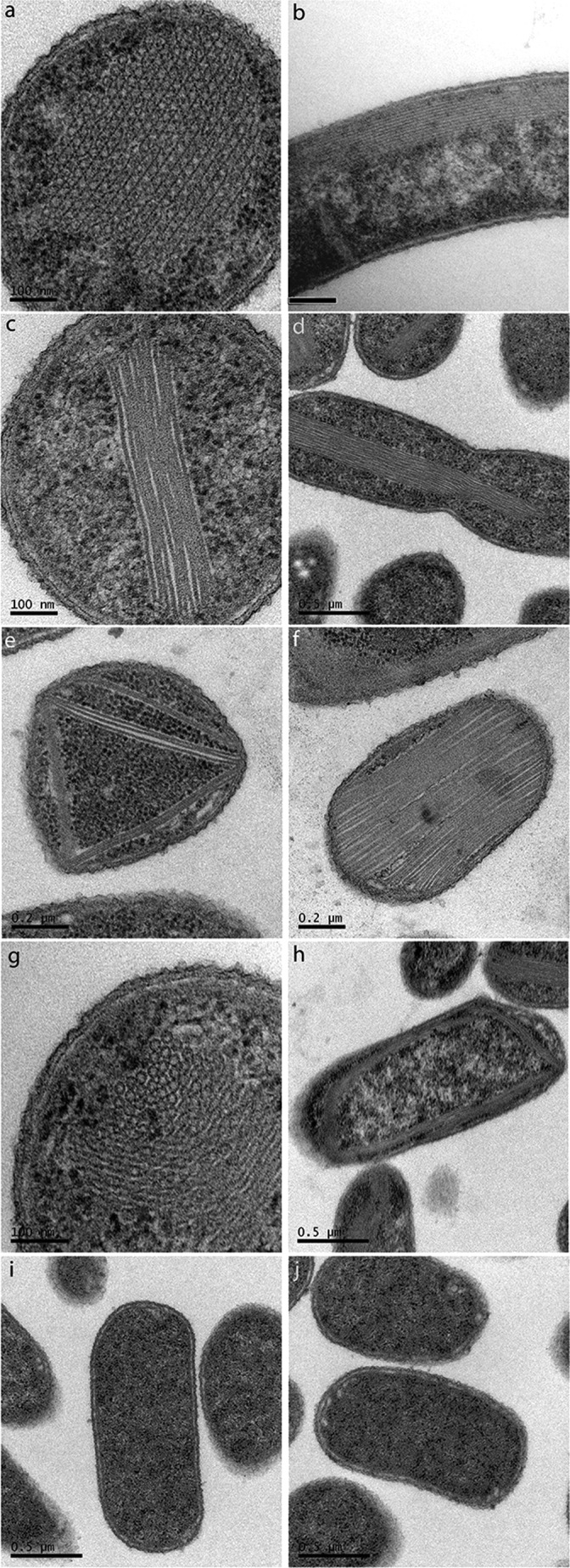
**Transmission electron microscopy of native PduA (*a* and *b*) and hexamer-hexamer interaction mutants (*c–j*) produced in *E. coli* cells.** Images on the *left* show transverse sections, and images on the *right* show longitudinal sections through the cell (except for the *last row*, where both are longitudinal sections). *a* and *b*, native PduA forms higher order structures that resemble honeycombs in transverse section (*a*) and tube-like structures in longitudinal sections (*b*). *c–h*, phenotypes of strains producing K26A (*c* and *d*), R79A (*e* and *f*), and V51A (*g* and *h*) interaction mutants. Both the K26A and the R79A mutant PduA micrographs show sheetlike assemblies that are layered. There is no evidence of PduA nanotubes for these mutants in transverse sections (*c* and *e*). The R79A PduA structures are more regularly packed than K26A PduA-derived structures. V51A PduA is the only mutant with different phenotypes in transverse (*g*) and longitudinal (*h*) view. V51A forms protein nanotubes of 18.3 ± 1.3-nm diameter (*e*), similar to the native PduA. No structures were observed for the K26D (*i*) or V51D (*j*) mutations. *Scale bar* in *b*, 0.5 μm; other *scale bars* can be seen more clearly in the *images*.

**FIGURE 3.**
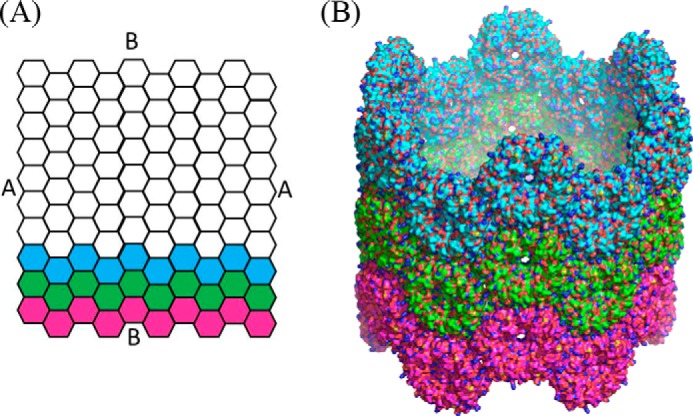
**A model of the native PduA-derived nanotubes.** *A*, a hexagonal sheet of PduA molecules can be rolled into a tube in one of two ways; either *A* can be connected to *A*, or *B* can be connected to *B*. In the model shown in *B*, the sheet is rolled so that *A* and *A* are brought together, and there are 12 hexamers per ring of the protein nanotube. The packing of hexamers within the ring and between rings is equivalent.

We have cultured, embedded, and analyzed an *E. coli* strain overproducing PduA using transmission electron microscopy. In agreement with previous observations (17), we observed that PduA gives rise to higher order structures that resemble honeycombs in transverse sections (Fig. 2*a*) and tubes in longitudinal sections (Fig. 2*b*). Measurement of the structures formed by PduA reveals that the structures are 20.4 ± 1.1 nm in diameter (20 measurements) and as long as the bacterial cell (1–2 μm). The honeycomb appearance in cross-section is suggestive of a bundle of tubular structures with a tendency to close hexagonal packing (Fig. 2*a*). We suggest that these filamentous structures are nanotubes. The lack of electron density in the lumen of the nanotubes is consistent with this hypothesis (it should be noted that a combination of positive (osmium) and negative (uranyl) staining has occurred during sample preparation). When viewed in longitudinal section, the tubes appear as pairs of lines corresponding to the negatively stained walls of the tubes (Fig. 2*b*). We propose that in the cytoplasm, the PduA molecules will tend to form a hexagonally tiled sheet similar to that seen in the crystal lattice for native protein (17). This sheet could form a tube by rolling up so that the outward facing vertex of one edge of the sheet could bind to the inward facing vertex of the other edge of the sheet (*A* to *A* in Fig. 3*A*). The need to interdigitate the vertices constrains the number of hexamers per turn to be even. Alternatively, the edges of the hexagons could meet (*B* to *B* in Fig. 3*A*).

Model building studies with the edges of the PduA hexamers perpendicular to the tube axis (*A* to *A* in Fig. 3*A*) revealed that to produce a tube consistent with the experimentally determined diameter of 20 nm required 12 hexamers (72 subunits) per turn (Fig. 3*B*). Because the hexamers are very stable, and the hexamer-hexamer junctions are far less so, we argue that it is the angle between adjacent hexamers that will change to accommodate the curvature to bring the first and 12th hexamers together. In fact, 12 hexamers per turn require an internal angle of 150°; the required curvature is therefore achieved by tilting succeeding hexamers through 30°. This is surprisingly readily achieved because the hexamers are wedge-shaped when viewed perpendicular to the 6-fold axis (Fig. 1*B*). In fact, the key interaction between the hexamers involving lysine 26 is maintained when adjacent hexamers are placed together at a 30° angle. No distortion of the hexamer is needed; nor are there any interpenetrating surfaces. The alternative way of rolling up the sheet, with hexamer edges approximately parallel to the tube axis and preserving the antiparallel hydrogen bonding of adjacent lysine 26 residues, produces a helix of 10 PduA hexamers per turn, of 20-nm diameter and with a pitch of two hexamers (138 Å). This structure can therefore be described as a two-start helix, each of pitch 138 Å. The precise arrangement of the hexamers within the nanotube remains to be resolved, but the important characteristics of both models are that the interhexamer interactions are preserved, the bending of adjacent hexamers is readily accommodated, and the concave surface of the hexamer faces outward and the convex surface faces the lumen of the tube so that the outside surface resembles the texture of a golf ball.

##### Analysis of the Native PduA Hexamer-Hexamer Interface

**FIGURE 4.**
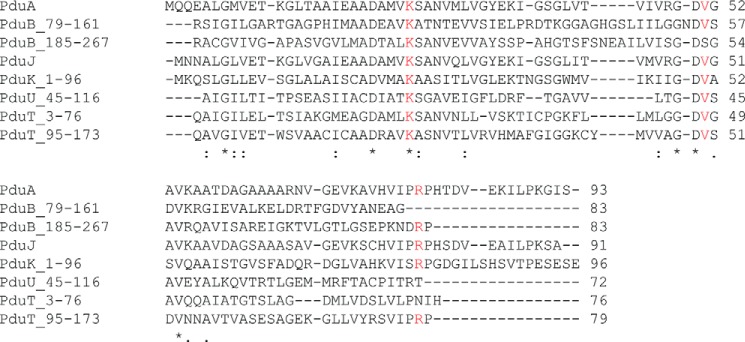
**Sequence alignment of shell proteins from *C. freundii* (PduA, PduB, PduJ, PduK, PduU, and PduT) using ClustalW2.** PduB and PduT were aligned using their individual domains determined by InterProScan4; where a subset of residues was used, the range is indicated. Residue conservation is indicated by an *asterisk*, and similarity is indicated by a *colon* or *period*. Residues selected for mutation are in *red*.

To probe our tube model, we aimed to disrupt the hexagonal tiling of PduA seen in the native crystal lattice and explore the effect this has on the formation of higher order structures visible in the bacterial cell cytoplasm. Calculation of changes in solvent accessibility when native PduA hexamers are brought together to form a sheet of molecules reveals residues that are at the interface (data not shown, but readily calculated using methods under “Experimental Procedures”). Lys-26 and Arg-79 have the highest solvent accessibility changes (77 and 139 Å^2^, respectively) and would therefore be expected to be of greatest importance for tiling in crystals and in microcompartment facets. Arg-79 can be clearly seen protruding from the isolated hexamer in Fig. 1*C*; it is positioned to plug into the adjacent hexamer in the sheet. Val-51 (57-Å^2^ solvent accessibility change) also occurs at the hexamer-hexamer interface, and its mutation was thought to influence the stability of hexamer-hexamer interactions although not as profoundly as Lys-26 and Arg-79. Substitutions planned were either replacements by alanine (truncation of the side chain) or by aspartate, which would be most perturbing to the interface when the substitution is close to the 2-fold axis between hexamers, bringing the substituted residues close together. Lys-26 to Asp was therefore anticipated to be most disruptive because the aspartates introduced would be proximal in the absence of structural rearrangement (Fig. 1*D*), whereas the substitution of Val-51 for Asp was anticipated to be least disruptive because this residue is further from the 2-fold axis relating adjacent hexamers, and therefore aspartate and its symmetry mate would not be in close proximity (Fig. 1*D*). Lysine 26 is involved in a characteristic antiparallel interaction, a hydrogen bond, with the 2-fold related hexamer (side-chain NZ to main-chain carbonyl; Fig. 1*D*) and is conserved across the shell proteins (Fig. 4).

##### Transmission Electron Microscopy Observations of the PduA Mutants

To study the effect the amino acid substitutions have on the phenotype of the tube-like structures, *E. coli* strains producing the Lys-26, Val-51, and Arg-79 mutants were cultured, embedded, sectioned, and visualized under the transmission electron microscope. Micrographs of strains producing the alanine mutants K26A and R79A show higher order structures, which appear to be multiple parallel sheets of proteins (Fig. 2, *c–f*). We suggest that these sheets are assembled from PduA hexamers in the bacterial cytoplasm. Multiple units of R79A-derived sheets were observed in a single cell, giving rise to striking arrangements that span the cell and even seem to stretch cells, causing shape changes (Fig. 2*e*). The V51A mutant is the only variant that forms structures with a distinct appearance in longitudinal and transverse (Fig. 2, *g* and *h*) section. In cross-section, PduA V51A appears to form honeycomb-like structures of 18.3 ± 1.3 nm in diameter (40 measurements). They are less regularly packed but of similar diameter (within error) to the structures seen previously with native PduA (20.4 ± 1.1 nm). Overexpression of K26D or V51D mutant PduAs resulted in no higher order structures being observed by TEM in the bacterial cytoplasm (Fig. 2, *i* and *j*) despite the proteins being produced in quantities suitable for crystallization and structure determination (see results below).

##### Crystal Structures of the PduA K26D Mutant

**FIGURE 5.**
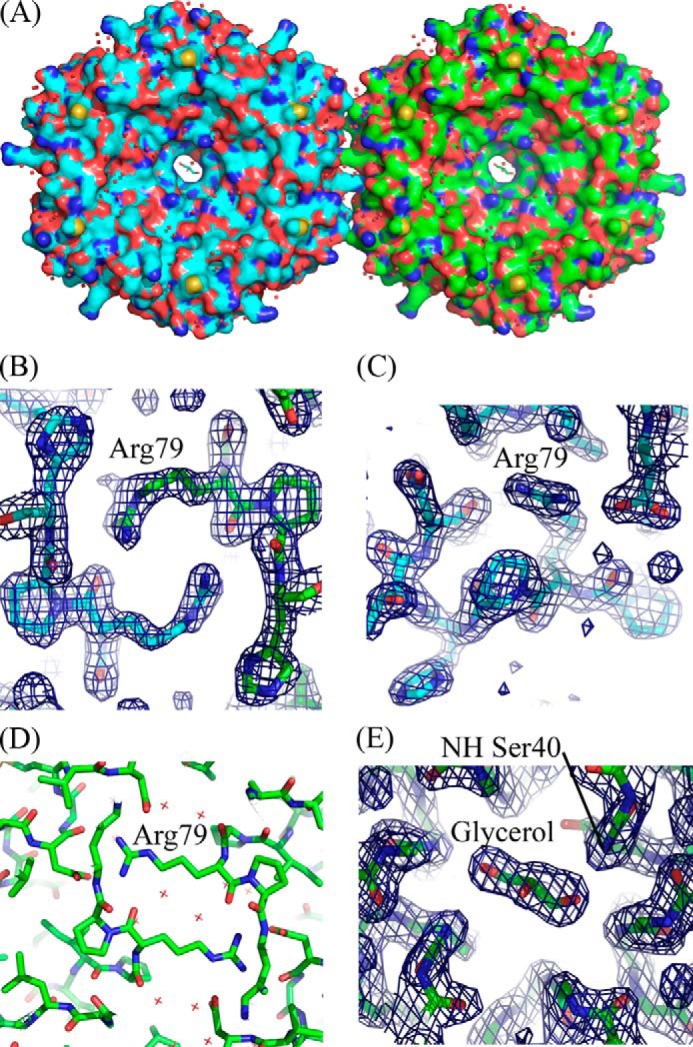
**Crystal structure of the K26D PduA mutant.** *A*, the strict 6-fold symmetry is lost in the K26D PduA mutant structure (type I crystals, 1.72 Å resolution), as can be best illustrated by examining Arg-79, which no longer has the regularity that can be seen for native PduA in Fig. 1*C*. The interface is also more fragile. *B*, the interaction between side-by-side hexamers is now dominated by Arg-79, which forms an intermolecular hydrogen bond with the main-chain carbonyl of Arg-79 from the hexamer with which it packs, which is surprisingly reminiscent of the lysine to main-chain carbonyl seen in the native structure. Asp-26 makes intermolecular hydrogen bonds to NE of Arg-79 (not shown). *C*, Arg-79 in subunit F, however, is involved in intrasubunit hydrogen bonds to OE1 of conserved Asp-22 and main-chain carbonyls of residues 83 and 84. Although adjacent hexamers can pack, as illustrated in *B*, they fail to tile in the P2_1_ crystal lattice. σ_A_-weighted 2*F*_obs_ − *F*_calc_ electron density is shown as *blue chicken wire mesh. D*, extended antiparallel arginine residues hydrogen-bonding to both aspartates 22 and 26 as well as the carbonyl of symmetry-related Arg-79 at the hexamer-hexamer interface. *E*, a single glycerol molecule (an analog of 1,2-propanediol substrate) is seen in the central pore of the hexamer.

The lysine 26 mutant is the most important to study structurally because Lys-26 is conserved in all shell proteins (Fig. 4) and contributes significantly to the interface between 2-fold related hexamers in the native crystals. Two crystal forms were obtained for this mutant using commercially available screens. There is clear electron density for residues 4–89 in nine copies of the PduA subunit across the two crystal forms, and the C-terminal residues, including the additional 23 residues, are not seen in the electron density maps. The fewest residues are seen for subunit C in type I crystals, where only residues 6–79 are clearly defined. In type I crystals, the K26D PduA hexamers form long strips of molecules in the P2_1_ lattice (Table 1) (*i.e.* the interaction shown in Fig. 5*A*) are repeated in one direction only (one-dimensional tiling in the diagonal of the *ac* plane) with a hexamer separation in the tiling distance of 67.4 Å, just slightly greater than the native distance of 67.2 Å. The strips of K26D PduA hexamers then pack alternately back to face to form the three-dimensional crystal lattice. The formation of strips is understandable when the conformation of Arg-79 is considered. Along the axis forming the strip, Arg-79 interacts with a 2-fold related hexamer (Fig. 5*B*) to form intermolecular hydrogen bonds; Arg-79 NE hydrogen-bonds to Asp-26′, and Arg-79 NH1 and NH2 hydrogen-bond the main-chain carbonyl of Arg-79′, but this interaction is not preserved at all potential hexamer-hexamer interfaces. This difference in conformation is most pronounced for the adjacent interface, where Arg-79 is involved in intramolecular interactions (C-subunit mentioned earlier). Here, Arg-79 hydrogen-bonds to Asp-22 and main-chain carbonyls of residues 83 and 84 (Fig. 5*C*).

**TABLE 1:** **Crystallographic data and refinement statistics for the K26D PduA mutant structure**

Crystal	Type I	Type II
Space group	P2_1_	P2_1_
Subunits in asymmetric unit	6	6
Cell parameters (Å)	45.2, 93.3, 63.1, β = 105.0°	68.0, 53.3, 68.1, β = 117.5°
Resolution range (Å)	60.89–1.72 (1.76–1.72)*^a^*	60.35–1.93 (2.0–1.93)
Observed reflections	269,196 (18,852)	75,170 (5,639)
No. of unique reflections	53,279 (3,903)	29,930 (2,254)
Completeness (%)	99.4 (98.6)	94.3 (95.5)
Multiplicity	5.1 (4.8)	2.5 (2.5)
〈*I*/σ(*I*)〉	13.9 (2.2)	10.7 (2.4)
*R*_merge_ (%)*^b^*	0.06 (0.617)	0.05 (0.363)
*R*-work/*R*-free	0.183/0.230	0.198/0.257
RMSD*^c^* (bonds) (Å)/RMSD angle (degrees)	0.026/2.507	0.016/1.750
Wilson *B*-factor (Å^2^)	29.1	32.9
No. of protein atoms	3,520	3,636
No. of water molecules	318	180/1 glycerol
Ramachandran plot statistics: residues in most favored/additional regions (%)	98/2	97/3

*^a^* The highest resolution range and parameters for that range are presented in parentheses.

*^b^ R*_merge_ = Σ*_hkl_* Σ*_i_* |*I_i_*(*hkl*) − (*I*(*hkl*))|/Σ*_hkl_* Σ*i I_i_*(*hkl*), where *I_i_*(*hkl*) is the *i*th observation of reflection *hkl*, and 〈*I*(*hkl*)〉 is the weighted average intensity for all observations of reflection *hkl*.

*^c^* RMSD, root mean square deviation.

In contrast, in the type II P2_1_ lattice, the K26D PduA hexamers do tile (two-dimensional tiling), but the separation of hexamers is greater than seen for native PduA at 68.0 and 68.1 Å along *a* and *c*, respectively, compared with 67.2 Å for native PduA, resulting in an interface that is clearly not as tight (Fig. 5*A*) as in the native PduA sheets (Fig. 1*A*). Remarkably, Arg-79 is now interacting with its 2-fold related symmetry mate in an elongated conformation reminiscent of the lysine interaction seen in native PduA (Fig. 5*D*). Here, the guanidinium group of Arg-79 is hydrogen-bonding to Asp-26′ (Arg-79 NE), Asp-22′ (Arg-79 NH_2_), and the carbonyl-oxygen atoms of Asp-22′ and Arg-79′ (the prime indicates a 2-fold related residue).

In both crystal forms, the regularity of the tiling is lost in that the K26D PduA hexamer no longer has strict 6-fold symmetry, and the separation between hexamers is greater than in native PduA sheets (67.2 Å). In the first form, strips of hexamers are formed, and in the second, sheets with rather poor hexamer-hexamer interactions are seen. It is perhaps not surprising then that no structures are seen for the K26D mutant in the bacterial cytoplasm when PduA K26D is overexpressed. A question of the effect of crystallization conditions arises because the conditions used were different from those used for crystallization of the *Salmonella enterica* PduA, but there is no obvious reason why the crystallization conditions would result in molecular asymmetry, whereas hexamer association is an observable cause of asymmetry. The breaking of the 6-fold symmetry allows a single glycerol molecule to be seen occupying the central pore of the hexamer (type II crystals; Fig. 5*E*). The O1 and O3 hydroxyls of this glycerol molecule hydrogen-bond to the main-chain amides of Ser-40 of subunits on opposite sides of the hexamer axis. The separation is 9.9 Å along this long axis as opposed to 8.5 Å for the axis at 60°.

## DISCUSSION

We investigate the effect of mutation of three residues identified as important in the association of hexamers on nanotube formation in the bacterial cytoplasm and, for a key mutation, K26D, the effect also on the packing of the hexamers in the crystal lattice (Table 2). Aspartate substitutions destabilize higher order structures to the extent that none are seen in TEM. This is consistent with the hexamer-hexamer interface seen in the crystal being important in the formation of the structures seen in TEM. For one of these mutants (K26D), crystals were obtained, and these crystals reveal more fragile hexamer-hexamer tiling than in native PduA in one crystal form (type II) and only one-dimensional tiling in the other (type I). The mutation K26D has a profound influence on the conformation of the neighboring Arg-79, such that this region of the protein no longer adopts a single conformation in the crystal, resulting in breaking of the 6-fold symmetry axis of the hexamer. This results in the imperfect tiling of PduA molecules seen in the crystals and apparent lack of tiling at all in the bacterial cytoplasm. The flexibility of the main-chain conformation around Arg-79 is a result of its position after the last β-strand of the bacterial microcompartment domain in a loop before the last α-helix, which tolerates structural change without affecting subunit folding or hexamer formation. But the influence of this residue on hexamer symmetry and tiling is profound.

**TABLE 2:**
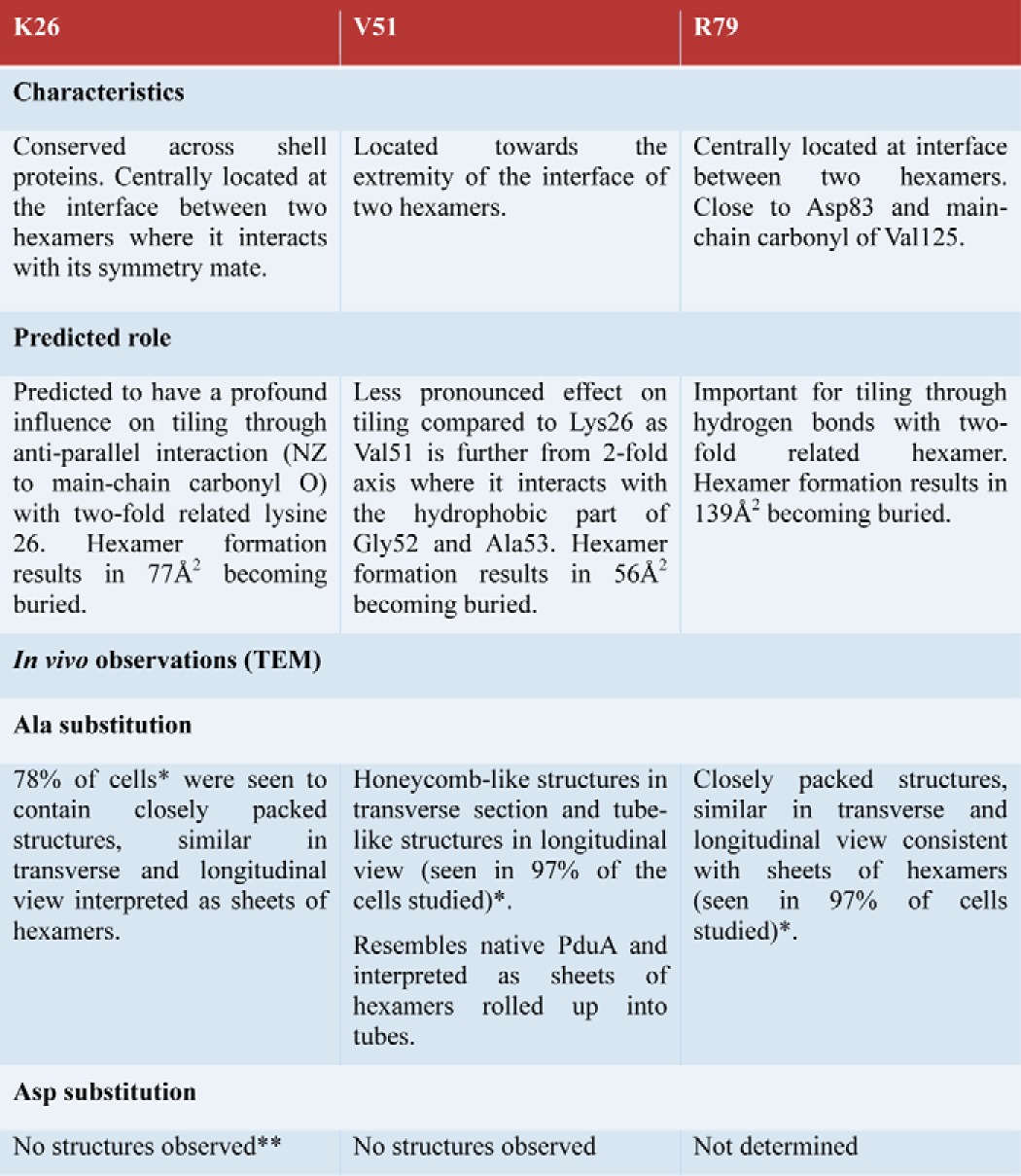
**Summary of PduA hexamer interaction mutants and TEM observations**

*, 200 cells were examined in detail for structures made by PduA and mutants of PduA; 100% of PduA cells examined showed tube-like structures. The other percentages given represent a minimum because it is possible that the structures were present but did not extend throughout the bacterial cell.

**, Two crystal forms were obtained, the regularity of the tiling is lost, and there is a breaking of strict 6-fold symmetry axis of hexamer.

The more modest alanine substitutions allow the formation of structures that appear similar in longitudinal and transverse section. We suggest that these are sheets of molecules in the bacterial cytoplasm. Only the V51A mutant gives tube-like structures resembling those formed using PduA. This suggests that the lysine 26 and arginine 79 interactions are profoundly important in maintaining the integrity of the molecular assembly as the hexamer-hexamer angle is increased from 0° (tiling a plane) to 30° (an angle necessary to form a tube with 12 hexamers per turn), and these interactions are preserved in our model of the nanotube. Overall, the results support the view that tiling of the microcompartment facet is due to interactions seen in the native PduA crystal lattice. They also suggest a mechanism by which the edges of the microcompartment could be formed where adjacent hexamers can associate at an out-of-plane angle of up to 30°. We have presented a model for the molecular architecture that underpins the nanotubes seen in TEM with PduA and V51A PduA. In addition, we have observed impressive arrangements of PduA structures that distort the cell's shape and segregate regions within the cell, confining ribosomes to certain areas. It may be possible to take advantage of these properties to permit the design of specific protein scaffolds or use them for controlled exclusion of certain regions of the bacterial cell.

Our crystallization studies have allowed us to see the first substrate analog (glycerol is closely similar to 1,2-propanediol) bound to the central pore of PduA (in the K26D mutant, which lacks 6-fold symmetry in the crystal lattice of the type II crystals), supporting the view that substrate can gain access to the microcompartment via the central pore of PduA. Compared with the long subunit pore described previously for PduB, which accommodates three glycerol molecules (31), the central pore within PduA is thin and binds a single glycerol. The contacts to the three glycerol molecules in PduB mainly involve side chains; here at the central pore, the contact is with the main-chain amide of Ser-40 from two PduA subunits. The presence of glycerol within the central channel, with coordinating loops distorted from precise 6-fold symmetry, suggests that variations from strict 6-fold symmetry seen are important for the biological function of PduA. Such insights can help in the redesign of shell protein pores for the passage of different metabolites.

In summary, structural insights into PduA have revealed how knowledge of shell proteins can be used to help the construction of large semipermeable protein scaffolds that could be employed for the targeted localization of specific pathways. Moreover, the detail on substrate binding to the pores within the shell protein can be used for the redesign of substrate entry/product release from bacterial microcompartments, which hold significant biotechnological potential for the incorporation of pathways with toxic intermediates (32).

## Footnotes

5 The abbreviations used are:

BMC: bacterial microcompartment
TEM: transmission electron microscopy
Pdu: 1,2-propanediol utilization.

## Note Added in Proof

Bobik and Yeates have recently published a study of residues important for the assembly of the microcompartment shell (Sinha, S., Cheng, S., Sung, Y. W., McNamara, D. E., Sawaya, M. R., Yeates, T. O., and Bobik, T. A. (2014) Alanine scanning mutagenesis identifies an asparagine-arginine-lysine triad essential to the assembly of the shell of the pdu microcompartment. *J. Mol. Biol.* **426,** 2328–2345.
